# Traditional activities and general and mental health of adult Indigenous peoples living off-reserve in Canada

**DOI:** 10.3389/fpubh.2023.1273955

**Published:** 2024-01-24

**Authors:** Hallah Kassem, M. Anne Harris, Ruby Edwards-Wheesk, Eric N. Liberda

**Affiliations:** ^1^School of Occupational and Public Health, Toronto Metropolitan University, Toronto, ON, Canada; ^2^Department of Chief and Council, Fort Albany First Nation, Fort Albany, ON, Canada

**Keywords:** Indigenous health, mental health, public health, epidemiology, traditional Indigenous activities

## Abstract

**Introduction:**

We examined associations between traditional Indigenous activities and self-perceived general and mental health in adult Indigenous persons living off-reserve in Canada using the 2012 and 2017 Aboriginal Peoples Surveys (APS), the two most recent datasets. We utilized four traditional Indigenous activities including hunting, making clothes or footwear, making arts or crafts, and gathering wild plants to investigate these self-reported data.

**Methods:**

Data from 9,430 and 12,598 respondents from the 2012 and 2017 APS, respectively, who responded to 15 questions concerning traditional activities were assessed using multivariable logistic regression to produce odds ratios (OR) and 95% confidence intervals (CI). Covariates included age, sex, education-level, income-level, Indigenous identity, residential school connection, ability to speak an Indigenous language, smoking status, and alcohol consumption frequency.

**Results:**

Using the 2012 APS, clothes-making was associated with poor self-reported general (OR = 1.50, 95%CI: 1.12–1.99) and mental (OR = 1.59, 95%CI: 1.14–2.21) health. Hunting was associated with good mental health (OR = 0.71 95%CI: 0.56–0.93). Similarly, 2017 analyses found clothes-making associated with poor general health (OR = 1.25, 95%CI: 1.01–1.54), and hunting associated with good general (OR = 0.76, 95%CI: 0.64–0.89) and mental (OR = 0.69, 95%CI: 0.58–0.81) health. Artmaking was associated with poor general (OR = 1.37, 95%CI: 1.17–1.60) and mental (OR = 1.85, 95%CI: 1.58–2.17) health.

**Conclusion:**

Hunting had protective relationships with mental and general health, which may reflect benefits of participation or engagement of healthier individuals in this activity. Clothes-making and artmaking were associated with poor general and poor mental health, possibly representing reverse causation as these activities are often undertaken therapeutically. These findings have implications for future research, programs and policies concerning Indigenous health.

## 1 Introduction

The health of Indigenous peoples in Canada has been significantly impacted by colonization. These impacts range from epidemics of the foreign diseases that accompanied the first arrivals of Europeans, to present day generational health inequities resulting from systemic discrimination and colonial policies like the Indian Act and the Residential Schools system (1, 2). In the 1830s, colonizers dispossessed Indigenous peoples by forcing them onto reserve land to make way for European settlers (3). In 1876, shortly after the creation of the Dominion of Canada, the Indian Act was passed with the ultimate goal of European assimilation of Indigenous peoples with clauses, since removed, restricting movement of Indigenous peoples outside of reserves and outlawing Indigenous ceremonies (4, 5). This act remains largely unchanged today and regulates taxation, governance, and rights to land, and recognizes and affirms the rights of First Nations peoples' as independent groups (4–6). The federally funded and church run Residential School System, largely modeled after Egerton Ryerson's study of native education, sought to erase Indigenous cultures by forcefully taking Indigenous children, preventing them from speaking their languages and practicing their culture, and inculcate Eurocentric practices. Horrifically, these facilities had high rates of physical and sexual abuse, disease, and death with mass graves of children still being rediscovered (7).

In 1876, the Indian Act prohibited anyone from living on reserve who was not “Status Indian” which was defined as males of Indian blood and their children and wives (3, 8). This lumped many nations into one category of First Nations and excluded many Indigenous groups including Inuit and Métis peoples, giving them no legal right to reserve land (3, 8). Today, three groups of Indigenous peoples are recognized in the Canadian Constitution: First Nations, Inuit and Métis. In 2016, 40% of Status First Nations people lived on a reserve with 60% living off-reserve, that is, with primary residence anywhere in Canada outside of the eight census subdivision types legally affiliated with First Nations or Indian bands (9). Although these groups are all native to the land now known as Canada, each of these groups have unique histories, cultures, and spiritual beliefs (9, 10). The term “Indigenous peoples” has prevailed over “Aboriginal” in recent years as it more aptly acknowledges that there are many unique groups of Indigenous individuals (9–11). The population of Indigenous peoples represents about 5% of Canada's population, though has grown at a much faster rate (1.77 times from 2016–2021) than that of non-Indigenous peoples (10, 12).

As an effect of colonial and discriminatory policies, Indigenous populations face numerous health inequities in Canada including higher rates of suicide (13), chronic disease (14), and mortality (15, 16). Compared to non-indigenous peoples, Indigenous communities face consistently higher suicide rates, with Inuit communities suffering 9 times greater rates of suicide than non-Indigenous peoples (13). Indigenous peoples also experience poorer overall health (17) and reduced life-expectancy. Indigenous men and women living off-reserve in Canada have a shorter life expectancy (72.1 years and 77.7 years, respectively) compared to non-Indigenous men (76 years) and women (81.5 years) (16). Indigenous peoples living off-reserve also face higher risks of obesity (18), cardiovascular diseases, respiratory diseases, cancers (15), heavy alcohol consumption (18–20), and twice the prevalence of diabetes (16).

In pre-contact times, that is, pre-European settlement, oral history suggests that Indigenous peoples enjoyed good holistic health due to active lifestyles and healthy traditional diets (2). There is extensive evidence of positive associations between health and time spent outdoors while, for example, hunting, gathering wild plants, or participating in programs designed to encourage on-the-land activity (21–23). Traditional activities of making clothes and artmaking have also been associated with positive mental and physical effects and are frequently used as therapeutic tools (24, 25). A 2021 study in Iiyiyiu Aschii found that though worries about pollution lead to Cree adults drinking tap water less, time spent outdoors and practicing traditional activities was unaffected (26). This further substantiates the importance of traditional activities within Indigenous communities.

Traditional Indigenous perceptions of health are often supported by teachings from the Medicine Wheel methodology of traditional healing that all things are related to and interact with everything else (27). Thus, health includes balance of self with external components like food, water, and land (28) as well as connections to heritage and cultural identity (28, 29). Traditional healing practices were repressed by colonial authorities, with attempts to eradication traditional healing practices explicitly described in some missionary writings (2). Colonization has impaired use of Indigenous languages and participation in traditional activities (30). These losses inhibit self-determination in Indigenous populations which is essential in building the traditional sense of balanced wellbeing (28).

This article presents a first step to addressing questions stemming from consultations with community members from Fort Albany First Nation who were interested in health impacts of traditional activities. The objective of our study was to assess associations between participation in specific traditional activities and self-perceived general and mental health in Indigenous adults living off-reserve using the 2012 and 2017 Aboriginal Peoples Surveys (APS), the two most recent APS.

## 2 Methods

### 2.1 Data source

Statistics Canada conducts APS in 5-year intervals with the specific aim to improve the wellbeing of Aboriginal Peoples in Canada (31, 32). Using a cross-sectional design, we analyzed the datasets of the 2012 and 2017 APS, the two most recent available datasets. The APS surveyed persons aged 6 years and older in the 2012 APS, and 15 years and older in the 2017 APS. Participants were individuals who identified as First Nations living off-reserve, Métis, or Inuit; “Status Indian” (Registered or Treaty Indian as defined by the Indian Act of Canada); and/or “member of a First Nation or Indian Band” (31, 33). A total of 38,150 individuals of more than 50,000 persons were eligible to participate in the 2012 APS resulting in a response rate of 76.3%; 32,330 individuals of more than 43,000 persons were eligible to participate in the 2017 APS resulting similarly in a response rate of 75.2% (31, 33). Survey questions asked participants about their traditional activity participation, education, geography, self-perceived health status, self-perceived mental health status, support access, housing, income, employment, language, mobility, identity, food security, education, alcohol and drug use, and healthcare utilization (34, 35). Reponses are linked with Canada's census and National Household Survey for additional variables. Survey questions were administered by telephone or in-person interviews in the language of the participants' choice, with proxy reporting used in rare cases requiring translation, or the participant was unable to answer directly (31).

This 2012 and 2017 APS Public Use Microdata File (PUMF) were produced by Statistics Canada and released to Canadian academic institutions in 2015 and 2020, respectively (33, 36). In the 2012 APS, proxy reporting was used for most children aged 6 to 14 years, and for nearly half of those ages 15 to 17 years. The 2017 APS included participants 15 and older and accepted proxy reporting by parents and guardians for participants ages 15 to 17. Both APS only accepted non-proxy responses for mental health status, so youth below the age of 18 were systematically excluded from reporting their mental health status. Indeed, approximately 36% of 2012 respondents ages 15 to 18 were not asked to report their mental health status. Further, the age categories of both APS included a category for between ages 15–18. Resultingly, the responses of mental health status in those 18 and younger are not representative. Thus, inclusion in this study was limited to respondents 19 and older with valid responses for included variables. This study was deemed not to require ethics approval as the data is publicly available and anonymized.

### 2.2 Primary outcomes

Health status was assessed by responses to “In general, would you say your health is...” for general health status and “In general, would you say your mental health is…[,]” for mental health; possible responses were “Excellent,” “Very Good,” “Good,” “Fair,” and “Poor.” These responses were dichotomized following a previously used and validated method (37–40). Responses were collapsed to Good (which included “Excellent,” “Very Good” and “Good”), and Poor (which included “Fair” and “Poor”) for comparison with common groupings in other APS studies (40–45). Self-perceived health status accounts for individually held values and relevant influences including age, sex, and socio-demographic characteristics (46). Self-perception also accounts for factors that are difficult to quantify, including disease severity and psychosocial effects, and is known to be an accurate and reliable tool in measuring health (46, 47).

### 2.3 Primary exposures

Four independent variables were used in analyzing individuals' participation (yes/no) in traditional activities: hunting, gathering wild plants, making arts and crafts, and making clothes and footwear. These categories were selected to align with the APS's description of traditional activities, described within the survey question asking participants whether or not they had done any traditional activities in the past year (31).

### 2.4 Covariates

#### 2.4.1 Sociodemographic and health indicators

Based on a priori findings demonstrating associations to self-perceived general and mental health, covariates included age, sex, education, income, identity, residential school attendance, language spoken, smoking, and alcohol use (42, 44, 48). Sex was reported dichotomously (male and female) in the 2012 APS and was reported as “Male,” “Female,” “Valid Skip,” “Don't Know,” “Refusal,” and “Not Stated” in the 2017 APS. However, all 2017 responses were either “Male” or “Female” thus only responses of “Male” or “Female” were included in analyses. Education was trichotomized as “less than high school,” “high school or equivalent,” and “more than high school” to correspond with similar studies (42, 44, 49). The seven APS levels of income were collapsed to four: “ < $10,000,” “between $10,000–$30,000,” “between $30,000–$50,000,” and “$50,000 and more” in the previous year to align with Statistics Canada's 2011 *Low-Income Cut-Offs* (50).

Participants were asked if they smoked cigarettes “daily,” “occasionally,” or “not at all.” We dichotomized smoking to current and non-smokers, in accordance with Ryan et al. (44). In the 2012 APS, alcohol consumption was defined through query of: “How often in the past 12 months have you had five or more drinks on one occasion.” In the 2017 APS, the corresponding question asked: “How often in the past 12 months have you had [five/four] or more drinks on one occasion.” We combined responses with a previous query on any alcohol consumption to create a variable indicating frequency of drinking 5 or more drinks in a single occasion in past year: “no alcohol use,” “never 5 or more drinks,” “5 or more drinks less than once a month,” “5 or more drinks once or more times per month.”

#### 2.4.2 Identity and culture

Participants identified as: First Nations (North American Indian), Inuk (Inuit) and/or Métis. Given the pervasive impacts of Canada's residential school system on health and culture of Aboriginal peoples (51), we included as a covariate respondents' experience with residential school, including federal industrial schools. Response options were: “Respondent attended,” “Only parents/grandparents attended,” “Only other family members attended,” “Only parents/grandparents/other family members attended,” “Neither respondent nor family attended.” As done previously (27), responses 2–4 were combined into “Only family members attended.” In the 2012 APS, Indigenous language denotes respondents' ability to speak a few words in an Indigenous language, and ability to speak or understand an indigenous language for the 2017 APS.

### 2.5 Statistical analyses

Descriptive statistics were presented as percentages for categorical variables. To account for the complexity of the sampling design, weights from the PUMF (33, 36) were used. Categorical bivariate associations with mental and general self-perception were examined within survey questions using *p-*values ascertained from chi square tests.

Associations were examined by modeling odds ratios (OR) using survey-weighted generalized linear models (SWGLM) with a logit link (logistic regression). A total of 95% confidence intervals (CI) were generated to assess error on point estimates. CIs not including 1 were considered “statistically significant” at alpha = 0.05. We constructed single, fully adjusted models for mental and general health, with participation in traditional activities as primary outcome variables, adjusted for age, sex, education, household income, alcohol use, smoking, residential school association, Indigenous language, and Indigenous identity for the 2012 and 2017 datasets separately. All analyses were conducted using R (v3.6.1) (52) and RStudio (v1.2.1335) (53). Where appropriate, *p*-values < 0.05 were regarded as statistically significant after Holms-Bonferroni adjustment.

### 2.6 Exclusions

Responses of “don't know,” “refusal,” “not stated,” or “valid skip” were excluded since these would prevent assessment of the factor. However, “valid skip” responses for level of alcohol consumption were included to ensure inclusion of respondents previously reporting not having had alcohol in the past year. Additionally, the APS only allowed non-proxy responses for mental health, therefore proxy responses were excluded.

## 3 Results

### 3.1 Descriptive results

**Figure 1:**
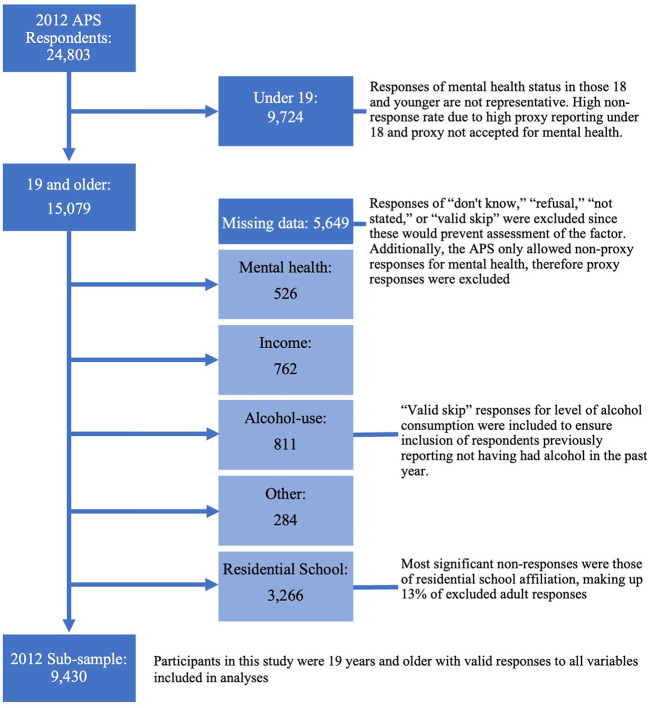
Flow diagram of participants excluded due to non-response in the 2012 APS sub-sample, created using sankeymatic.com.

The 2012 APS PUMF included 24,803 respondents, of whom 15,079 were 19 and older. Approximately 3% of participants refused queries on general health and 3.5% refused queries on mental health. In total, approximately 37.5% of adult respondents were excluded from the sample due to incomplete data, most (3,266) due to incomplete responses regarding residential school attendance. As demonstrated in Figure 1, the analytic subsample included 9,430 respondents.

**Figure 2:**
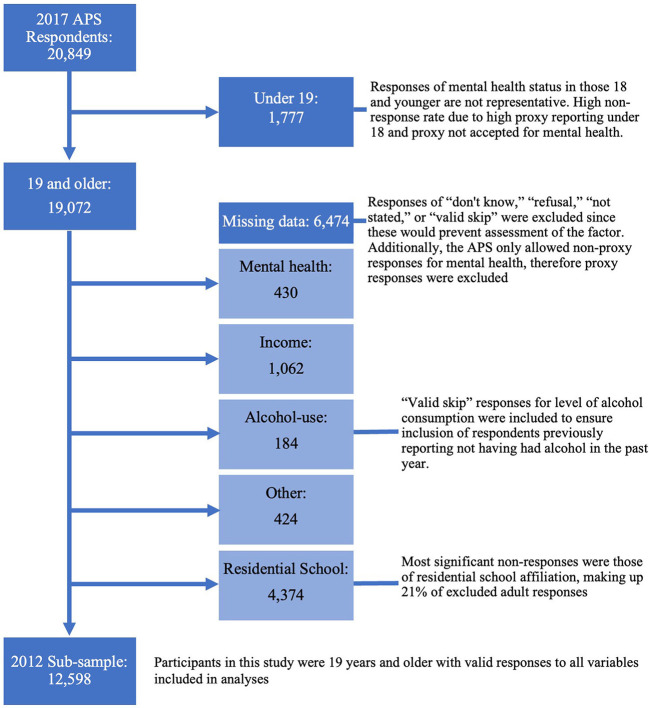
Flow diagram of participants excluded due to non-response in the 2017 APS sub-sample, created using sankeymatic.com.

The 2017 APS PUMF contained data from 20,849 respondents, of whom 19,072 were 19 and older. Less than 0.2% of participants refused queries on general health and roughly 5% refused queries on mental health. Approximately 31.7% of respondents ages 19 and older were ineligible for selection in the sample due to incomplete data, again, most drastically due to residential school attendance responses (4,374). This analytic subsample included 12,598 respondents, demonstrated in Figure 2.

Tables 1, 2 present demographics distributions and bivariate associations. Good general health was reported by 81.9% and 80.6% of 2012 and 2017 APS respondents, respectively, and 89.9% and 85.5% reported good mental health, respectively. Bivariate analyses of both 2012 and 2017 APS data indicated associations between clothes-making and poor general and mental health, and between artmaking and poor mental health. Protective associations were found between hunting and good general and mental health, in both surveys. Incongruently, gathering plants was associated with poor general health in only the 2012 APS; and artmaking was associated with poor general health in only the 2017 APS.

**Table 1:** Descriptive statistics and bivariate associations with general and mental health in 9,430 participants in the 2012 APS.

	**General health**					**Mental health**				
	**Good (*****N** =* **7,724)**		**Poor (*****N** =* **1,706)**		***p*-value**	**Good (*****N** =* **8,478)**		**Poor (*****N** =* **952)**		***p*-value**
	**Proportion of N**	**n**	**Proportion of N**	**n**		**Proportion of N**	**n**	**Proportion of N**	**n**	
**Health outcomes**										
Good general health	—	—	—	—	—	84.3%	332,256	34.8%	17,656	**< 0.001**
Good mental health	95.0%	332,256	65.2%	62,006	**< 0.001**	—	—	—	—	—
**Traditional activities**										
Makes clothes	8.9%	185,074	15.7%	41,991	**< 0.001**	9.5%	204,012	16.6%	23,052	**< 0.001**
Gathers wild plants	31.8%	144,433	36.2%	43,513	**< 0.05**	32.4%	165,051	35.0%	22,896	0.35
Hunts	38.7%	159,096	29.5%	33,868	**< 0.001**	37.7%	175,381	28.9%	17,583	**< 0.001**
Makes arts and crafts	27.9%	126,645	30.7%	40,705	0.17	27.6%	145,852	35.5%	21,499	**< 0.01**
**Age**										
19–24	15.6%	54,554	7.2%	6,814	**< 0.001**	13.9%	54,694	13.2%	6,674	0.41
25–34	21.8%	76,367	13.1%	12,424		20.2%	79,535	18.3%	9,255	
35–44	22.1%	77,404	18.2%	17,335		21.0%	82,640	23.9%	12,099	
45–54	20.5%	71,870	24.6%	23,389		21.1%	83,239	23.7%	12,020	
55+	19.9%	69,718	36.9%	35,074		23.9%	94,154	21.0%	10,637	
**Sex**										
Female	54.5%	252,384	64.4%	65,851	**< 0.001**	55.5%	285,535	65.3%	32,700	**< 0.001**
Male	45.5%	97,528	35.6%	29,184		44.5%	108,727	34.7%	17,986	
**Education**										
< High school	17.4%	318,815	33.6%	80,123	**< 0.001**	19.7%	356,681	29.9%	42,256	**< 0.001**
High school	15.7%	31,097	13.9%	14,913		15.1%	37,581	17.0%	8,429	
>High school	66.9%	60,812	52.5%	31,972		65.2%	77,632	53.1%	15,151	
**Household income**										
< $9,999	14.5%	54,931	21.9%	13,202	**< 0.001**	15.3%	59,505	22.5%	8,628	**< 0.001**
$10,000–$29,999	30.3%	234,169	52.4%	49,862		33.2%	257,125	49.3%	26,906	
$30,000–$49,999	24.2%	173,692	13.9%	54,342		22.9%	199,708	15.3%	28,326	
$50,000+	30.9%	158,490	11.8%	36,777		28.6%	175,524	13.0%	19,744	
**5**+ **Alcoholic drinks**										
No alcohol	16.9%	96,752	34.8%	18,284	**< 0.001**	19.5%	103,725	30.2%	11,311	**< 0.001**
Never	27.3%	98,603	25.7%	19,187		27.8%	104,078	20.4%	13,712	
Less than once/month	27.7%	222,900	19.2%	43,781		26.3%	245,927	22.3%	20,754	
Once or more/month	28.2%	127,012	20.2%	51,255		26.4%	148,335	27.1%	29,932	
**Smoking status**										
Current smoker	36.3%	95,573	53.9%	24,460	**< 0.001**	37.6%	109,686	59.1%	10,347	**< 0.001**
**Residential school**										
No attendance	52.9%	50,751	44.2%	20,835	**< 0.001**	51.7%	60,180	45.5%	11,406	**< 0.05**
Respondent attended	5.8%	106,117	10.0%	49,773		6.4%	130,925	9.3%	24,965	
Family attended	41.3%	84,754	45.8%	13,248		41.9%	90,259	45.2%	7,744	
**Ability to speak an Indigenous language**										
Speak Indigenous language	36.2%	238,796	42.8%	60,590	**< 0.01**	37.0%	266,431	42.4%	32,955	**< 0.05**
**Identity**										
First nations	49.6%	15,112	57.2%	3,253	**< 0.01**	50.7%	16,461	55.9%	1,905	0.07
Métis	45.3%	2,618	38.7%	663		44.5%	2,570	39.0%	711	
Inuit	4.3%	214,612	3.4%	66,991		4.2%	245,575	3.8%	36,028	
Multiple	0.7%	135,300	0.7%	28,044		0.7%	148,687	1.4%	14,658	

Participants were 19 years and older with valid responses to the variables listed. Bivariate associations between each variable and general and mental health were assessed with chi-square analyses and resulting *p*-values. The *p*-values in bold indicate statistical significance. APS, Aboriginal Peoples Survey; N, sample size; n, weighted sample size.

**Table 2:** Descriptive statistics and bivariate associations with general and mental health in 12,598 participants in the 2017 APS.

	**General health**					**Mental health**				
	**Good (*****N** =* **10,148)**		**Poor (*****N** =* **2,450)**			**Good (*****N** =* **10,770)**		**Poor (*****N** =* **1,828)**		
	**Proportion of N**	**n**		**Proportion of N**	**n**		**Proportion of N**	**n**		**Proportion of N**
**Health outcomes**										
Good general health	—	—	—	—	—	86.7%	430,540	41.5%	37,491	**< 0.001**
Good mental health	92.0%	430,540	55.6%	66,002	**< 0.001**	—	—	—	—	—
**Traditional activities**										
Makes clothes	9.2%	250,979	12.7%	47,929	**< 0.001**	9.3%	261,590	13.2%	37,318	**< 0.001**
Gathers wild plants	32.2%	193,300	32.3%	58,951	0.94	32.3%	205,112	31.9%	47,139	**< 0.001**
hunts	39.7%	220,521	29.2%	50,651	**< 0.001**	39.3%	238,718	27.9%	32,453	**< 0.001**
Makes arts and crafts	22.1%	186,687	27.5%	56,397	**< 0.001**	21.0%	203,749	35.4%	39,335	**< 0.001**
**Age**										
19–24	15.3%	71,592	10.1%	12,019	**< 0.001**	13.5%	66,889	18.5%	16,722	**< 0.001**
25–34	21.2%	99,339	13.5%	16,009		19.4%	96,248	21.2%	19,100	
35–44	19.4%	90,933	14.6%	17,378		18.4%	91,600	18.5%	16,711	
45–54	19.9%	92,917	25.1%	29,796		20.9%	103,688	21.1%	19,025	
55+	24.2%	113,250	36.7%	43,609		27.8%	138,117	20.8%	18,742	
**Sex**										
Female	52.9%	364,422	57.4%	86,120	**< 0.01**	51.9%	392,181	64.1%	58,362	**< 0.001**
Male	47.1%	103,608	42.6%	32,692		48.1%	104,361	35.9%	31,939	
**Education**										
< High school	14.1%	424,957	24.8%	103,692	**< 0.001**	15.9%	450,308	17.9%	78,341	0.31
High school	15.0%	43,074	14.4%	15,120		14.8%	46,234	15.3%	11,960	
>High school	70.9%	65,785	60.8%	29,498		69.3%	79,086	66.8%	16,197	
**Household income**										
< $9,999	11.0%	70,294	19.7%	17,070	**< 0.001**	11.6%	73,587	19.3%	13,776	**< 0.001**
$10,000–$29,999	29.6%	331,952	47.1%	72,244		30.6%	343,868	47.4%	60,328	
$30,000–$49,999	22.1%	223,078	17.8%	63,812		22.1%	240,125	16.6%	46,766	
$50,000+	37.2%	220,051	15.4%	48,744		35.7%	228,946	16.7%	39,850	
**5**+ **Alcoholic drinks**										
No alcohol	17.9%	140,508	32.9%	24,502	**< 0.001**	20.5%	142,443	23.5%	22,567	0.08
Never	23.6%	133,240	26.1%	24,243		24.0%	133,218	24.6%	24,265	
Less than once/month	30.0%	320,509	20.6%	64,219		28.7%	334,817	25.0%	49,912	
Once or more/month	28.5%	147,521	20.4%	54,592		26.8%	161,724	26.9%	40,389	
**Smoking status**										
Current smoker	31.5%	110,337	45.9%	30,976	**< 0.001**	32.6%	119,109	44.7%	22,204	**< 0.001**
**Residential school**										
No attendance	53.6%	51,485	40.3%	23,443	**< 0.001**	52.7%	57,472	41.3%	17,457	**< 0.001**
Respondent attended	5.1%	138,712	10.0%	55,922		6.0%	151,864	6.5%	42,770	
Family attended	41.3%	103,596	49.6%	21,134		41.3%	109,707	52.2%	15,023	
**Ability to speak an Indigenous language**										
Speak Indigenous language	39.9%	317,241	47.5%	80,397	**< 0.001**	41.0%	336,142	43.6%	61,495	0.17
**Identity**										
First nations	47.7%	20,141	53.7%	5,007	**< 0.001**	48.4%	22,107	51.8%	3,040	**< 0.05**
Métis	47.0%	4,761	41.0%	1,248		46.1%	5,364	44.1%	645	
Inuit	4.3%	282,405	4.2%	84,146		4.5%	301,417	3.4%	65,134	
Multiple	1.0%	185,626	1.1%	34,666		1.1%	195,125	0.7%	25,167	

Participants were 19 years and older with valid responses to the variables listed. Bivariate associations between each variable and general and mental health were assessed with chi-square analyses and resulting *p-*values. The *p*-values in bold indicate statistical significance. APS, Aboriginal Peoples Survey; N, sample size; n, weighted sample size.

The characteristics of participants who participated in each traditional activity are presented in Tables 3, 4. In both surveys, participants who reported gathering wild plants or making clothes were older than those who participated in making art or hunting. Participants of any traditional activity were mostly female, except those of hunting for which 59% of participants were male. Similarly, though similar distributions of income were observed for participants in all other traditional activities, those who participated in hunting had higher average incomes. Additionally, 86.9% and 88.9% of respondents who participated in making clothes were female in the 2012 and 2017 APS, respectively. In the 2012 APS, 50% or more of those who participated in making art, gathering plants, or making clothes reported never having consumed alcohol in the past year or never having consumed 5 or more drinks in past year; in the 2017 APS this was only observed for participants who reported making clothes.

**Table 3:** Descriptive statistics and bivariate associations with participation in traditional activities in 9,430 participants in the 2012 APS.

	**Makes arts and crafts**			**Hunts**			**Gathers wild plants**			**Makes clothes**		
	***N** =* **126,712**			***N** =* **163,345**			***N** =* **145,562**			***N** =* **46,010**		
	**n'**	**Proportion of n**	***p*-values**	**n'**	**Proportion of n**	***p*-values**	**n'**	**Proportion of n**	***p*-values**	**n'**	**Proportion of n**	***p*-values**
**Health outcomes**										
Good general	97,528	77.0%	0.17	135,300	82.8%	**< 0.001**	111,116	76.3%	**< 0.05**	31,097	67.6%	**< 0.001**
Poor general	29,184	23.0%		28,044	17.2%		34,446	23.7%		14,913	32.4%	
Good mental	108,727	85.8%	**< 0.01**	148,687	91.0%	**< 0.001**	127,831	87.8%	0.35	37,581	81.7%	**< 0.001**
Poor mental	1,7986	14.2%		14,658	90.0%		17,730	12.2%		8,429	18.3%	
**Traditional activities**										
Makes clothes	28,882	22.8%	**< 0.001**	18,938	12.0%	0.06	28,351	19.5%	**< 0.001**	—	—	**< 0.001**
Gathers wild plants	66,661	52.6%	**< 0.001**	76,464	46.8%	**< 0.001**	—	—	**< 0.001**	28,351	61.6%	**< 0.001**
Hunts	47,284	37.3%	0.64	—	—	**< 0.001**	76,464	52.5%	**< 0.001**	18,938	41.2%	0.06
Makes arts and crafts	—	—	**< 0.001**	47,284	29.0%	0.64	66,661	45.8%	**< 0.001**	28,882	62.8%	**< 0.001**
**Age**										
19–24	19,969	15.8%	**< 0.001**	24,667	15.1%	**< 0.001**	14,965	10.3%	**< 0.001**	5,356	11.6%	**< 0.05**
25–34	27,827	22.0%		37,430	22.9%		27,552	18.9%		7,980	17.3%	
35–44	29,735	23.5%		37,401	22.9%		31,818	21.9%		8,424	18.3%	
45–54	26,920	21.2%		32,466	19.9%		33,657	23.1%		11,496	25.0%	
55+	22,261	17.6%		31,380	19.2%		37,569	25.8%		12,754	27.7%	
**Sex**										
Female	84,867	67.0%	**< 0.001**	67,679	41.4%	**< 0.001**	89,552	61.5%	**< 0.001**	39,983	86.9%	**< 0.001**
Male	41,845	33.0%		95,665	58.6%		56,010	38.5%		6,027	13.1%	
**Education**										
< High school	20,025	15.8%	**< 0.001**	33,821	20.7%	0.55	27,560	18.9%	**< 0.01**	8,710	18.9%	0.64
High school	16,867	13.3%		23,772	14.6%		19,178	13.2%		7,260	15.8%	
>High school	89,821	70.9%		105,752	64.7%		98,824	67.9%		30,040	65.3%	
**Household income**										
< $9,999	22,555	17.8%	**< 0.001**	23,348	14.3%	**< 0.001**	23,330	16.0%	0.09	9,335	20.3%	**< 0.001**
$10,000–$29,999	49,778	39.3%		48,143	29.5%		53,269	36.6%		20,451	44.4%	
$30,000–$49,999	26,364	20.8%		35,217	21.6%		28,581	19.6%		8,690	18.9%	
$50,000+	28,015	22.1%		56,637	34.7%		40,382	27.7%		7,534	16.4%	
**5**+ **Alcoholic drinks**										
No alcohol	24,686	19.5%	**< 0.05**	25,602	15.7%	**< 0.001**	29,466	20.2%	**< 0.001**	12,222	26.6%	**< 0.001**
Never	37,958	30.0%		38,580	23.6%		47,849	32.9%		16,748	36.4%	
Less than once/month	34,223	27.0%		44,149	27.0%		36,389	25.0%		9,799	21.3%	
Once or more/month	29,845	23.6%		55,014	33.7%		31,858	21.9%		7,241	15.7%	
**Smoking status**										
Current smoker	53,876	42.5%	0.07	70,007	42.9%	**< 0.01**	60,886	41.8%	0.13	19,555	42.5%	0.33
**Residential school**										
No attendance	57,966	45.7%	**< 0.001**	80,061	49.0%	**< 0.05**	64,418	44.3%	**< 0.001**	17,587	38.2%	**< 0.001**
Respondent attended	8,863	7.0%		10,016	6.1%		11,078	7.6%		4,736	10.3%	
Family attended	59,884	47.3%		73,267	44.9%		70,065	48.1%		23,687	51.5%	
**Ability to speak an Indigenous language**										
Speaks Indigenous language	58,066	45.8%	**< 0.001**	72,398	44.3%	**< 0.001**	70,671	48.6%	**< 0.001**	24,935	54.2%	**< 0.001**
**Identity**										
First nations	65,840	52.0%	0.1	77,315	47.3%	**< 0.001**	72,791	50.0%	**< 0.001**	22,700	49.3%	**< 0.001**
Métis	55,613	43.9%		72,666	44.5%		62,775	43.1%		17,214	37.4%	
Inuit	3,957	3.1%		11,649	7.1%		8,752	6.0%		5,308	11.5%	
Multiple	1,303	1.0%		1,715	1.0%		1,243	0.9%		788	1.7%	

Participants were 19 years and older with valid responses to the variables listed. Bivariate associations between each variable and participation in traditional activities were assessed with chi-square analyses and resulting *p*-values. The *p*-values in bold indicate statistical significance. APS, Aboriginal Peoples Survey; N, sample size; n, weighted sample size, n', weighted sub-sample size.

**Table 4:** Descriptive statistics and bivariate associations with participation in traditional activities in 12,598 participants in the 2017 APS.

	**Makes arts and crafts**			**Hunts**			**Gathers wild plants**			**Makes clothes**		
	***N** =* **136,300**			***N** =* **220,291**			***N** =* **189,205**			***N** =* **58,194**		
	**n'**	**Proportion of n**	***p*-values**	**n'**	**Proportion of n**	***p*-values**	**n'**	**Proportion of n**	***p*-values**	**n'**	**Proportion of n**	***p*-values**
**Health outcomes**												
Good general health	103,608	76.0%	**< 0.001**	185,626	84.3%	**< 0.001**	150,790	79.7%	0.94	43,074	74.0%	**< 0.001**
Poor general health	32,692	24.0%		34,666	15.7%		38,415	20.3%		15,120	26.0%	
Good mental health	104,361	76.6%	**< 0.001**	195,125	88.6%	**< 0.001**	160,399	84.8%	0.81	46,234	79.4%	**< 0.001**
Poor mental health	31,939	23.4%		25,167	11.4%		28,805	15.2%		11,960	20.6%	
**Traditional activities**												
Makes clothes	30,266	22.2%	**< 0.001**	23,918	10.9%	**< 0.05**	35,765	18.9%	**< 0.001**		0.0%	**< 0.001**
Gathers wild plants	66,452	48.8%	**< 0.001**	106,834	48.5%	**< 0.001**		0.0%	**< 0.001**	35,765	61.5%	**< 0.001**
Hunts	54,186	39.8%	**< 0.05**		0.0%	**< 0.001**	106,834	56.5%	**< 0.001**	23,918	41.1%	**< 0.05**
Makes arts and crafts		0.0%	**< 0.001**	54,186	24.6%	**< 0.05**	66,452	35.1%	**< 0.001**	30,266	52.0%	**< 0.001**
**Age**												
19–24	24,202	17.8%	**< 0.001**	29,690	13.5%	**< 0.001**	18,324	9.7%	**< 0.001**	5,680	9.8%	**< 0.001**
25–34	31,789	23.3%		43,923	19.9%		32,359	17.1%		11,929	20.5%	
35–44	26,265	19.3%		48,946	22.2%		43,432	23.0%		13,768	23.7%	
45–54	24,877	18.3%		47,288	21.5%		41,679	22.0%		10,459	18.0%	
55+	29,167	21.4%		50,444	22.9%		53,411	28.2%		16,357	28.1%	
**Sex**												
Female	89,749	65.8%	**< 0.001**	89,481	40.6%	**< 0.001**	107,466	56.8%	**< 0.01**	51,742	88.9%	**< 0.001**
Male	46,551	34.2%		130,810	59.4%		81,739	43.2%		6,452	11.1%	
**Education**												
< High school	16,472	12.1%	**< 0.001**	31,991	14.5%	**< 0.05**	25,396	13.4%	**< 0.001**	7,203	12.4%	**< 0.001**
High school	18,506	13.6%		33,158	15.1%		22,536	11.9%		6,483	11.1%	
>High school	101,321	74.3%		155,143	70.4%		141,274	74.7%		44,508	76.5%	
**Household income**												
< $9,999	19,675	14.4%	**< 0.001**	21,901	9.9%	**< 0.001**	21,526	11.4%	**< 0.001**	9,668	16.6%	**< 0.001**
$10,000–$29,999	51,498	37.8%		57,030	25.9%		58,687	31.0%		21,337	36.7%	
$30,000–$49,999	29,943	22.0%		48,510	22.0%		40,592	21.5%		12,889	22.1%	
$50,000+	35,184	25.8%		92,849	42.1%		68,400	36.2%		14,300	24.6%	
**5**+ **Alcoholic drinks**												
No alcohol	27,285	20.0%	0.1	36,788	16.7%	**< 0.001**	38,988	20.6%	**< 0.05**	15,301	26.3%	**< 0.001**
Never	34,159	25.1%		47,950	21.8%		49,772	26.3%		16,754	28.8%	
Less than once/month	40,794	29.9%		65,985	30.0%		52,786	27.9%		16,209	27.9%	
Once or more/month	34,061	25.0%		69,568	31.6%		47,659	25.2%		9,930	17.1%	
**Smoking status**												
Current Smoker	48,241	35.4%	0.39	77,992	35.4%	0.24	63,480	33.6%	0.32	19,659	33.8%	0.71
**Residential school**												
No attendance	63,978	46.9%	**< 0.001**	112,472	51.1%	0.79	85,726	45.3%	**< 0.001**	22,872	39.3%	**< 0.001**
Respondent attended	7,077	5.2%		12,782	5.8%		13,057	6.9%		4,398	7.6%	
Family attended	65,244	47.9%		95,037	43.1%		90,422	47.8%		30,923	53.1%	
**Ability to speak an Indigenous language**												
Speaks Indigenous language	65,325	47.9%	**< 0.001**	102,339	46.5%	**< 0.001**	102,213	54.0%	**< 0.001**	35,478	61.0%	
**Identity**												
First nations	70,338	51.6%	**< 0.01**	103,602	47.0%	**< 0.001**	96,763	51.1%	**< 0.001**	29,371	50.5%	**< 0.001**
Métis	59,368	43.6%		100,378	45.6%		78,848	41.7%		21,106	36.3%	
Inuit	4,737	3.5%		14,753	6.7%		11,397	6.0%		7,058	12.1%	
Multiple	1,857	1.4%		1,558	0.7%		2,198	1.2%		658	1.1%	

Participants were 19 years and older with valid responses to the variables listed. Bivariate associations between each variable and participation in traditional activities were assessed with chi-square analyses and resulting *p-*values. The *p*-values in bold indicate statistical significance. APS, Aboriginal Peoples Survey; N, sample size; n, weighted sample size; n', weighted sub-sample size.

### 3.2 Analytic results

In both surveys, most demographic, health and Indigenous cultural factors were associated with general and mental health in bivariate analyses (Tables 1, 2). Poor general health was associated with older age, female sex, less education, lower income, less alcohol consumption, smoking, and experience with residential school. Poor mental health showed similar associations as general health with the exception of a null association with education and alcohol consumption in the 2012 APS, and age in the 2017 APS. Relationships with Indigenous language and identity were also observed but are complex to interpret given potential relationships to sociodemographic factors. Bivariate results must be interpreted with caution due to possible confounding.

In fully adjusted models, participating in traditional activities within the past year showed relationships with both mental and general health (Table 5). In both surveys, making clothes was associated with poor general health, and hunting was associated with good mental health. Making clothes was also associated with poor mental health in the 2012 APS. In the 2017 APS, artmaking was associated with poor general and poor mental health, and hunting was associated with good general health. Slight increases in odds of good general and mental health were found in the 2017 APS compared to the 2012 APS for participation in making clothes, gathering plants, and hunting. The association between making clothes and mental health was attenuated in the 2017 survey compared to the 2012 survey. The associations between making arts and crafts was strengthened in the 2017 compared to the 2012 survey.

**Table 5:** Results of multivariable logistic regression models of poor general health and poor mental health and associations with participation in traditional activities.

	**2012 APS (*****N** =* **9,430)**				**2017 APS (*****N** =* **12,598)**			
	**Poor general health**		**Poor mental health**		**Poor general health**		**Poor mental health**	
	**OR**	**95%CI**	**OR**	**95%CI**	**OR**	**95%CI**	**OR**	**95%CI**
Makes clothes	**1.5**	**1.13–1.99**	**1.6**	**1.14–2.21**	**1.3**	**1.01–1.54**	1.1	0.89–1.41
Gathers wild plants	1.1	0.88–1.31	1.1	0.83–1.35	1.0	0.84–1.16	1.0	0.83–1.16
Hunts	**0.8**	**0.66–1.00**	**0.7**	**0.56–0.93**	**0.8**	**0.64–0.89**	**0.7**	**0.58–0.81**
Makes arts or crafts	1.1	0.84–1.31	1.2	0.94–1.65	**1.4**	**1.17–1.60**	**1.9**	**1.58–2.17**

Each model adjusted for traditional activities, age, sex, education, household income, alcohol use, smoking, residential school, indigenous language, and indigenous identity. APS, Aboriginal Peoples Survey; CI, confidence interval; OR, odds ratio; N, sample size. Values in bold text indicate statistical significant.

Sensitivity analyses with and without self-perceived mental health in modeled poor general health (and vice-versa) did not significantly alter the observed relationships with traditional activities. Since 13% and 21% of excluded adult responses in the 2012 and 2017 APS, respectively, were due to residential school responses, sensitivity analyses were conducted to assess the effect of this restriction. Unrestricted responses did not alter direction or significance of most results; however, inclusion of non-responses resulted in loss of significance of associations between making clothes with mental health [1.3 (0.97, 1.74)] in the 2012 APS and with general health [1.2 (0.96, 1.40)] in the 2017 APS. Further, in comparing the age, sex and income distributions between the non-response participants to those who responded, no significant differences were observed.

## 4 Discussion

### 4.1 Interpretation

Our findings require careful interpretation in the context of these cross-sectional analyses. Several of the covariate factors showed relationships to health in bivariate analyses. As in previous analyses of the APS (51), experiences with residential schools were associated with poorer general and mental health. Kaspar also reported the observation shown in our bivariate analyses of the inverse association between Inuit identity and poor health (51). These findings must not be misinterpreted as a comparison to the general Canadian population. Inuit populations in Canada face significant food insecurity (54), poor water quality (55, 56), and other health challenges (57).

Hunting is an important aspect of Indigenous culture with positive impacts on mental health (58). Though there lacks recent studies of hunting and health in Indigenous populations in Canada, hunting shows complex relationships to health in Indigenous peoples globally (28, 55). In Indigenous communities, consumption of “country food” has been linked with physical and psychological benefits (59), and increased physical activity from hunting-and-gathering lifestyles has been linked to cardiovascular health benefits (60). Interpretation of the benefits of hunting is complicated by the physical demands of hunting, which may bar those experiencing impaired general or mental health; this is analogous to the healthy worker effect. Although, anecdotally, many Indigenous persons perform on-the-land activities such as hunting and trapping to improve wellbeing (21). In fact, we have recently shown that on-reserve Indigenous activities are associated with improved biophysical responses such as increased omega-3 polyunsaturated fats (21).

A related phenomenon could explain associations of making clothes and artmaking with poor general and mental health. We expected these activities to offer health protection, as in prior assessments in other cultures. One study found associations between knitting and reduced mild cognitive impairment in the United States (61), and a Norwegian study found artistic cultural activities was associated with improved health and lower levels of depression and anxiety (62). A 2021 South African study found fashion could help women with disabilities manage low-self-esteem (63). Conversely, a similar study using the 2012 APS found traditional activity participation increased odds of anxiety by 46%, though researchers noted this may be due to some participation being a form of therapy (48). We, too, note potential reverse causation or confounding by indication (64): making clothes and artmaking are often used as therapy, thus participants of poor health may be more likely to participate. Further, the low demands of these activities may be favored by those unable to engage in physically demanding activities such as hunting. Anecdotally, our team member, Ruby Edwards-Wheesk, observed this within her own First Nations reserve community, especially among ages who find it difficult to hunt (2021, personal conversation). Residual confounding by age and income could also affect these unexpected relationships since the APS groupings are coarse and within-group variability cannot be measured; this is relevant if older adults or those of low-income are systematically more likely to make clothes and make art. It is complex to interpret differences in strengths of these associations between the 2012 and 2017 surveys; one possibility is underlying periodic trends in these cultural practices and which specific activities fall under these categorizations. We did note that self-reported overall participation in making clothes decreased between the 2012 and 2017 cycles, and participation in making arts and crafts increased. Additional community-based research to explore specific traditional activities, the populations engaged by these activities, and their relative impacts before and after participation, is needed.

### 4.2 Limitations

The key limitation of using the APS is its cross-sectional design which prevents causal determination. Also, since APS results are only made available 3 years after data collection, the results of analyses using the APS may not accurately represent current relationships. That being said, the data from the 2017 APS used herein is the most up-to-date data available. Additionally, since no universal scale of self-perceived health exists it is difficult to make accurate cross-cultural comparisons (46). Since the APS includes only those living off-reserve these results are not generalizable to those living on-reserve. The APS did not distinguish between social and non-social participation which could affect health outcomes, especially in older adults (65). In fact, loneliness has been shown to be associated with decreased cognitive function (65), vision loss (66), and heart disease and stroke (67).

One notable limitation of this study is the absence of data concerning the intensity of the primary exposure, i.e., engagement in traditional activities. While the APS provides an overview of whether respondents partake in traditional activities, it lacks granularity in terms of frequency, duration, and depth of engagement. This omission hampers our ability to draw nuanced conclusions about dose-response relationships between traditional activities and health outcomes.

The study also does not account for the possible variations in the understanding and interpretation of terms related to traditional activities across different Indigenous groups. Terms like “traditional activities,” “mental health,” or “wellbeing” may have culturally specific meanings that are not captured in a one-size-fits-all survey instrument like the APS. This limitation could affect the generalizability of our findings to all Indigenous communities.

Another significant limitation is the lack of geographic and specific Indigenous grouping data in the APS dataset, which restricts our ability to conduct subgroup analyses. This is a critical shortcoming as health outcomes and engagement in traditional activities may vary significantly across regions and among different Indigenous groups. Without this data, the study's findings may not be fully representative or generalizable.

## 5 Conclusion

Given the findings of this study, there are several recommendations for future programs, plans, and policies aimed at improving the general and mental health of adult Indigenous persons living off-reserve in Canada. Firstly, there is a need for culturally sensitive interventions that recognize the importance of traditional activities in the health and wellbeing of Indigenous communities. Policymakers should consult with Indigenous leaders and organizations to design and implement such interventions. Next, considering the changes in findings between the first and second survey waves, it would be prudent to investigate potential environmental, social, or policy changes that may have occurred during this period. These changes could contribute to the observed differences in health outcomes and engagement in traditional activities.

The APS serves as a valuable tool for understanding the lives of on-reserve Indigenous peoples in Canada, but it could benefit from several improvements. The inclusion of questions that capture the intensity of engagement in traditional activities, as well as geographic and Indigenous subgroup data, would enhance its utility for research and policy. Additionally, making the APS dataset more readily available to researchers and policymakers could facilitate more extensive and varied analyses, ultimately contributing to better-informed decisions for Indigenous communities.

An important step in reconciling health inequities facing Indigenous communities is determining associations between traditional activities and health. Our results provide evidence of complex associations between practicing traditional activities and self-perceived general and mental health. These findings have important implications for future research of traditional activities and for the development of programs, plans, and policies affecting Indigenous populations in Canada.

## Data availability statement

The original contributions presented in the study are included in the article/supplementary material. The 2012 and 2017 APS data is accessible at Statistics Canada Research Data Centres (RDCs). The application to access the data is available at www.statcan.gc.ca/en/microdata/data-centres/access.

## Ethics statement

The studies involving humans were approved by the Health Canada, Public Health Agency of Canada (PHAC), and Research Ethics Board (REB). The studies were conducted in accordance with the local legislation and institutional requirements. Written informed consent for participation was not required from the participants or the participants' legal guardians/next of kin in accordance with the national legislation and institutional requirements.

## Author contributions

HK: Conceptualization, Formal analysis, Investigation, Visualization, Writing—original draft, Writing—review & editing. MH: Methodology, Supervision, Writing—review & editing. RE-W: Conceptualization, Writing—review & editing. EL: Conceptualization, Resources, Supervision, Writing—review & editing.

## References

[1] Immigration citizenship. Discover Canada - Canada's History. (2015). Available oline at: https://www.canada.ca/en/immigration-refugees-citizenship/corporate/publications-manuals/discover-canada/read-online/canadas-history.html (accessed November 8, 2023).

[2] First Nations Health Authority. Our History, Our Health. Available online at: https://www.fnha.ca/wellness/wellness-for-first-nations/our-history-our-health (accessed November 8, 2023).

[3] Facing History & Ourselves,. Dispossession, Destruction, the Reserves. (2020). Available oline at: https://www.facinghistory.org/en-ca/resource-library/dispossession-destruction-reserves (accessed November 13, 2023).

[4] First, Nations Studies Program. The Indian Act. Available oline at: http://indigenousfoundations.arts.ubc.ca/the_indian_act/ (accessed November 8, 2023).

[5] Government of Canada. The Indian Act. 1999 Canada.

[6] Facing History & Ourselves,. Historical Background: The Indian Act the Indian Residential Schools (2019). Available oline at: https://www.facinghistory.org/en-ca/resource-library/historical-background-indian-act-indian-residential-schools (accessed November 13, 2023).

[7] Facing History & Ourselves,. Killing the Indian in the Child. (2019). Available oline at: https://www.facinghistory.org/en-ca/resource-library/killing-indian-child (accessed November 25, 2023).

[8] Facing History Ourselves Canada. Defining the Indian. (2020). Available oline at: https://www.facinghistory.org/en-ca/resource-library/defining-indian (accessed November 13, 2023).

[9] Canada S. Indigenous Peoples Reference Guide Census of Population, 2021 (2022). Available online at: https://www12.statcan.gc.ca/census-recensement/2021/ref/98-500/009/98-500-x2021009-eng.cfm (accessed November 13, 2023).

[10] Crown-Indigenous Relations Northern Affairs Canada. Indigenous peoples and communities. (2022). Available oline at: https://www.rcaanc-cirnac.gc.ca/eng/1100100013785/1529102490303 (accessed November 13, 2023).

[11] Indigenous Corporate Training. Indigenous vs. Aboriginal. (2016). Available oline at: https://www.ictinc.ca/blog/indigenous-vs.-aboriginal (accessed November 25, 2023).

[12] Canada S. Canada's Indigenous population. StatsCAN Plus. (2023). Available oline at: https://www.statcan.gc.ca/o1/en/plus/3920-canadas-indigenous-population (accessed November 13, 2023).

[13] Kumar M Tjepkema M. Suicide among First Nations people, Métis and Inuit (2011–2016): Findings from the 2011 Canadian Census Health and Environment Cohort (CanCHEC). Consumer Policy Research Database (2019).

[14] Bruce SG Riediger ND Lix LM Bruce SG Riediger ND LL. Chronic disease and chronic disease risk factors among First Nations, Inuit and Métis populations of northern Canada. Chronic Dis Inj Can. (2014) 34:210–7. 10.24095/hpcdp.34.4.04 25408180

[15] Tjepkema M Wilkins R Senécal S Guimond É Penney C. Mortality of Métis and Registered Indian adults in Canada: an 11-year follow-up study. Stat Canada Heal Reports. (2009) 20:31–51. 20108604

[16] Frohlich KL Ross N Richmond C. Health disparities in Canada today: some evidence and a theoretical framework. Health Policy. (2006) 79:132–43. 10.1016/j.healthpol.2005.12.010 16519957

[17] Cooke M Mitrou F Lawrence D Guimond E Beavon D. Indigenous well-being in four countries: an application of the UNDP'S human development index to indigenous peoples in Australia, Canada, New Zealand, and the United States. BMC Int Health Hum Rights. (2007) 7:1–11. 10.1186/1472-698X-7-9 18096029 PMC2238768

[18] Gionet L Roshanafshar S. Select health indicators of First Nations people living off reserve, Métis Inuit. Statistics Canada Health at a Glance (2015). Available oline at: https://www150.statcan.gc.ca/n1/pub/82–624-x/2013001/article/11763-eng.htm (accessed May 19, 2021).

[19] Canada G of. A Statistical Profile on the Health of First Nations in Canada: Determinants of Health, 2006 to 2010. (2020). Available oline at: https://www.sac-isc.gc.ca/eng/1585414580249/1585414609942 (accessed May 19, 2021).

[20] Elton-Marshall T Leatherdale ST Burkhalter R. Tobacco, alcohol and illicit drug use among Aboriginal youth living off-reserve: results from the youth smoking survey. Can Med Assoc J. (2011) 183:E480–6. 10.1503/cmaj.101913 21555383 PMC3091934

[21] Moriarity RJ Zuk AM Liberda EN Tsuji LJS. Health measures of Eeyouch (Cree) who are eligible to participate in the on-the-land Income Security Program in Eeyou Istchee (northern Quebec, Canada). BMC Public Health. (2021) 21:1–11. 10.1186/s12889-021-10654-7 33789644 PMC8011104

[22] Liberda EN Zuk AM Martin ID Tsuji LJS. Fisher's linear discriminant function analysis and its potential utility as a tool for the assessment of health-and-wellness programs in indigenous communities. Int J Environ Res Public Health. (2020) 17:1–18. 10.3390/ijerph17217894 33126498 PMC7663610

[23] Tsuji LJS Tsuji SRJ Zuk AM Davey R Liberda EN. Harvest programs in first nations of subarctic canada: The benefits go beyond addressing food security and environmental sustainability issues. Int J Environ Res Public Health. (2020) 17:1–24. 10.3390/ijerph17218113 33153153 PMC7663715

[24] Camic PM. Playing in the mud: Health psychology, the arts and creative approaches to health care. J Health Psychol. (2008) 13:287–98. 10.1177/1359105307086698 18375633

[25] Csikszentmihalyi M. The Systems Model of Creativity the Collected Works of Mihaly Csikszentmihalyi. Dordrecht: Springer. (2014). 10.1007/978-94-017-9085-7

[26] Moriarity RJ Zuk AM Liberda EN Tsuji LJS. The self-reported behaviour of Iiyiyiu Aschii Cree and the worry about pollution from industrial and hydroelectric development in northern Quebec, Canada. Environ Res. (2021) 195:110788. 10.1016/j.envres.2021.110788 33508258

[27] Graham H Stamler LL. Contemporary perceptions of health from an indigenous (plains cree) perspective. Int J Indig Heal. (2010) 6:6–17. 10.18357/ijih61201012341

[28] King M Smith A Gracey M. Indigenous health part 2: the underlying causes of the health gap. Lancet. (2009) 374:76–85. 10.1016/S0140-6736(09)60827-8 19577696

[29] Stewart SL. Promoting Indigenous mental health: Cultural perspectives on healing from Native counsellors in Canada. Int J Heal Promot Educ. (2008) 46:49–56. 10.1080/14635240.2008.10708129

[30] Wilson K Rosenberg MW Abonyi S. Aboriginal peoples, health and healing approaches: the effects of age and place on health. Soc Sci Med. (2011) 72:355–64. 10.1016/j.socscimed.2010.09.022 21036444

[31] Cloutier E Langlet É. Aboriginal Peoples Survey, 2012: Concepts and Methods Guide. Canada: Statistique Canada. (2014).

[32] Statistics Canada. Surveys statistical programs - Aboriginal Peoples Survey (APS). (2018). Available oline at: https://www23.statcan.gc.ca/imdb/p2SV.pl?Function=getSurvey&SDDS=3250 (accessed July 1, 2020).

[33] Vongdara B Léger D Budinski R. Aboriginal Peoples Survey 2017: User's guide to the Public Use Microdata File (PUMF). Centre for Indigenous Statistics Partnerships (2020). Available online at: https://www150.statcan.gc.ca/n1/en/catalogue/89-653-X2020001

[34] Statistics Canada. Aboriginal Peoples Survey, 2012 [Canada] Study Documentation (2015). Available online at: https://www150.statcan.gc.ca/n1/en/catalogue/89-653-X2015005

[35] Statistics Canada. Aboriginal Peoples Survey 2017 Data Dictionary (2020). Available online at: https://www150.statcan.gc.ca/n1/daily-quotidien/200602/dq200602d-eng.htm

[36] Budinski R Langlet É. Aboriginal Peoples Survey 2012: User's Guide to the Public Use Microdata File (PUMF). (2015). Available online at: https://www150.statcan.gc.ca/n1/en/catalogue/89-653-X2015005

[37] Bougie E Arim RG Kohen DE Findlay LC. Validation of the 10-item kessler psychological distress scale (K10) in the 2012 aboriginal peoples survey. Stat Canada Heal Reports. (2016) 27:3–10. 26788720

[38] Rashidi A Higgs P Carruthers S. Aboriginal people with chronic HCV: The role of community health nurses for improving health-related quality of life. Collegian. (2019) 27:250–7. 10.1016/j.colegn.2019.08.006

[39] Manor O Matthews S Power C. Dichotomous or categorical response? Analysing self-rated health and lifetime social class. Int J Epidemiol. (2000) 29:149–57. 10.1093/ije/29.1.149 10750617

[40] Wilson K Rosenberg MW Abonyi S Lovelace R. Aging and health: an examination of differences between older aboriginal and non-aboriginal people ^*^. Can J Aging/La Rev Can du Vieil. (2010) 29:369–82. 10.1017/S0714980810000309 20731890

[41] Ali-Hassan H Eloulabi R Keethakumar A. Internet non-use among Canadian Indigenous older adults: aboriginal Peoples Survey (APS). BMC Public Health. (2020) 20:1–11. 10.1186/s12889-020-09659-5 33059658 PMC7560061

[42] Ryan CJ Cooke M Leatherdale ST. Factors associated with heavy drinking among off-reserve First Nations and Métis youth and adults: evidence from the 2012 Canadian Aboriginal Peoples Survey. Prev Med. (2016) 87:95–102. 10.1016/j.ypmed.2016.02.008 26861752

[43] Janz T Seto J Turner A. Aboriginal Peoples Survey, 2006: An Overview of the Health of the Métis Population. Statistics Canada, Social and Aboriginal Statistics Division. (2013).

[44] Ryan CJ Cooke MJ Leatherdale ST Kirkpatrick SI Wilk P. The correlates of current smoking among adult Métis: evidence from the Aboriginal Peoples Survey and métis supplement. Can J Public Heal. (2015) 106:e271–6. 10.17269/cjph.106.5053 26451987 PMC6972307

[45] Bethune R Absher N Obiagwu M Qarmout T Steeves M Yaghoubi M. Social determinants of self-reported health for Canada's indigenous peoples: a public health approach. Public Health. (2019) 176:172–180. 10.1016/j.puhe.2018.03.007 29666024

[46] Statistics Canada. Perceived health. Healthy people, healthy places Statistics Canada. (2016). Available oline at: https://www150.statcan.gc.ca/n1/pub/82–229-x/2009001/status/phx-eng.htm (accessed April 24, 2020).

[47] Short ME Goetzel RZ Pei X Tabrizi MJ Ozminkowski RJ Gibson TB. How accurate are self-reports? Analysis of self-reported health care utilization and absence when compared with administrative data. J Occup Environ Med. (2009) 51:786–96. 10.1097/JOM.0b013e3181a86671 19528832 PMC2745402

[48] Nasreen S Brar R Brar S Maltby A Wilk P. Are indigenous determinants of health associated with self-reported health professional-diagnosed anxiety disorders among canadian first nations adults? Findings from the 2012 aboriginal peoples survey. Community Ment Health J. (2017) 54:460–8. 10.1007/s10597-017-0165-0 28887731

[49] Bougie E Kohen D. Smoking among off-reserve first nations, métis, and inuit high school students. Int Indig Policy J. (2018) 9:7543. 10.18584/iipj.2018.9.2.1

[50] Statistics Canada. Low income cut-offs (LICOs) before and after tax by community size and family size, in current dollars. (2020). Available oline at: https://www150.statcan.gc.ca/t1/tbl1/en/tv.action?pid=1110024101 (accessed January 13, 2020).

[51] Kaspar V. The lifetime effect of residential school attendance on indigenous health status. Am J Public Health. (2014) 104:2184–90. 10.2105/AJPH.2013.301479 24328622 PMC4202939

[52] R Core Team. R software. R Foundation for Statistical Computing. (2008).

[53] Rstudio Team. RStudio: Integrated development for R. Boston MA: RStudio, Inc. (2019).

[54] Egeland GM Pacey A Cao Z Sobol I. Food insecurity among Inuit preschoolers: nunavut inuit child health survey, 2007–2008. Can Med Assoc J. (2010) 182:243–8. 10.1503/cmaj.091297 20100848 PMC2826465

[55] Daley K Castleden H Jamieson R Furgal C Ell L. Municipal water quantities and health in Nunavut households: an exploratory case study in Coral Harbour, Nunavut, Canada. Int J Circumpolar Health. (2014) 73:23843. 10.3402/ijch.v73.23843 24765615 PMC3970036

[56] Medeiros AS Wood P Wesche SD Bakaic M Peters JF. Water security for northern peoples: review of threats to Arctic freshwater systems in Nunavut, Canada. Reg Environ Chang. (2017) 17:635–47. 10.1007/s10113-016-1084-2

[57] Healey GK Meadows LM. Inuit women's health in Nunavut, Canada: a review of the literature. Int J Circumpolar Health. (2007) 66:199–214. 10.3402/ijch.v66i3.18256 17655061

[58] Samson C Pretty J. Environmental and health benefits of hunting lifestyles and diets for the Innu of Labrador. Food Policy. (2006) 31:528–53. 10.1016/j.foodpol.2006.02.001

[59] Condon RG Collings P Wenzel G. The best part of life : subsistence hunting, ethnicity, and economic adaptation among young adult inuit males. Arctic. (1995) 48:31–46. 10.14430/arctic1222

[60] Raichlen DA Pontzer H Harris JA Mabulla AZP Marlowe FW Josh Snodgrass J. Physical activity patterns and biomarkers of cardiovascular disease risk in hunter-gatherers. Am J Hum Biol. (2017) 29:e22919. 10.1002/ajhb.22919 27723159

[61] Geda YE Topazian HM Lewis RA Roberts RO Knopman DS Pankratz VS. Engaging in cognitive activities, aging, and mild cognitive impairment: a population-based study. J Neuropsychiatry Clin Neurosci. (2011) 23:149–54. 10.1176/jnp.23.2.jnp149 21677242 PMC3204924

[62] Cuypers K Krokstad S Lingaas Holmen T Skjei Knudtsen M Bygren LO Holmen J. Patterns of receptive and creative cultural activities and their association with perceived health, anxiety, depression and satisfaction with life among adults: the HUNT study, Norway. J Epidemiol Commun Health. (2012) 66:698–703. 10.1136/jech.2010.113571 21609946

[63] Kalitanyi V. Role of fashion as a form of therapy among women with disabilities in South African. J Bus Retail Manag Res. (2021) 16:14–23. 10.24052/JBRMR/V16IS01/ART-02

[64] Salas M Hofman A Ch Strieker BH. Confounding by indication: an example of variation in the use of epidemiologic terminology. Am J Epidemiol. (1999) 149:981–3. 10.1093/oxfordjournals.aje.a009758 10355372

[65] Shankar A Hamer M McMunn A Steptoe A. Relationships with cognitive function during 4 years of follow-up in the English longitudinal study of ageing. Psychosom Med. (2013) 75:161–70. 10.1097/PSY.0b013e31827f09cd 23362501

[66] Mick P Parfyonov M Wittich W Phillips N Pichora-Fuller MK. Associations between sensory loss and social networks, participation, support, and loneliness: Analysis of the Canadian Longitudinal Study on Aging. Can Fam Physician. (2018) 64:e33–41. 29358266 PMC5962968

[67] Holt-Lunstad J Smith TB Baker M Harris T Stephenson D. Loneliness and social isolation as risk factors for mortality. Perspect Psychol Sci. (2015) 10:227–37. 10.1177/1745691614568352 25910392

